# Deliberative process of health technology reassessment by health technology assessment agency in Korea

**DOI:** 10.1017/S026646232400014X

**Published:** 2024-05-13

**Authors:** Sangjin Shin, Yunjung Kim, Jieun Choi, Jung Yul Park

**Affiliations:** 1 Division of Healthcare Technology Assessment Research, National Evidence-based healthcare Collaborating Agency, Seoul, Republic of Korea; 2 Department of Neurosurgery, Korea University Anam Hospital, Korea University College of Medicine, Seoul, Republic of Korea

**Keywords:** health technology reassessment, technology management, health insurance, clinical safety, in-use technology, recommendation

## Abstract

In 2019, the National Evidence-based Healthcare Collaborating Agency (NECA) in Korea established a health technology reassessment (HTR) system to manage the life cycle of health technologies and develop operational measures promoting the efficient use of healthcare resources. The purpose of this study is to introduce the detailed implementation process and practical functional methods of the HTR implemented by NECA.

The HTR is a structured multidisciplinary method for analyzing health technologies currently used in the healthcare system based on the latest information on parameters, such as clinical safety, effectiveness, and cost-effectiveness of optimizing the use of healthcare resources as well as social and ethical issues. All decision-making stages of the HTR are carefully reviewed and transparently managed. The HTR committee makes significant decisions, and the subcommittee decides the details related to the assessment process.

Since the pilot began in 2018, 262 cases have been reassessed, of which, 126 cases (48.1 percent) were health services not covered by the National Health Insurance (NHI). Over the past 5 years, approximately 130 recommendations for the in-use technologies were determined by the HTR committee. In the near future, it will be necessary to officially develop and establish a Korean HTR system and a legal foundation to optimize the NHI system.

## Background

Health technology reassessment (HTR) is an emerging field that shifts focus from traditional methods of technology adoption to technology management throughout its lifecycle. HTR is defined as “a structured, evidence-based assessment of the clinical, social, ethical, and economic effects of a technology, currently used in the healthcare system, to inform optimal use of that technology in comparison to alternatives” (1–4). Therefore, HTR can be considered as a mechanism to improve patient care and system efficiency by reallocating resources from low-value care to higher-value interventions and technologies (5).

Factors affecting healthcare expenditure, including population aging and health technology advancement driven by the ongoing Fourth Industrial Revolution (6;7), continue to rise. Therefore, achieving balance between the fiscal soundness of health insurance and coverage expansion is a key factor. In the past 10 years, the National Health Insurance (NHI) coverage rate in Korea has stagnated at slightly above 60 percent and non-covered health services have increased. To reduce the burden of health expenses, all medically necessary non-covered existing items need to be included in NHI benefits. For items and services that have uncertain cost-effectiveness, the non-covered services and items will be designated to a new program called “Preliminary Benefit.” This program applies differentiated patient copayment rates such as 50, 80, and 90 percent. It is a transitional program to raise the NHI coverage rates in the current situation of prevalent non-covered services and items (8). After 3–5 years, a reassessment of these services and items determines their potential inclusion within the NHI. This policy underscores the need for additional management mechanisms to reassess existing health technologies with uncertain safety, effectiveness and cost-effectiveness. Following the announcement of the NHI coverage reinforcement policy in August 2017, the Ministry of Health and Welfare (MoHW) proposed to establish an HTR system to manage health technologies within the Medical Service Act framework. This system would aid in decision-making regarding the appropriateness of NHI benefits according to the newly organized NHI coverage categories.

Since the implementation of health technology assessment (HTA) in Korea, the NHI system has primarily focused on regulating the entry of new technologies through the HTA system. This includes establishing criteria for reimbursement of drug and medical procedures as mandated by the MoHW. However, currently, there is no standardized process for monitoring the ongoing utilization or managing the withdrawal of a technology within the NHI system, especially if a technology becomes obsolete due to knowledge advancement.

Since 2019, the National Evidence-based Healthcare Collaborating Agency (NECA), a national HTA agency in Korea, has established a surge in political demands. In response, NECA has established an HTR system to effectively manage the life cycle of existing health technologies. Furthermore, it has devised operational measures to optimize the efficient utilization of healthcare resources. As an independent HTA institution, NECA supports policy decision-making and improves policy acceptability through objective reviews of scientific evidence, adhering to the standardized operating procedure. In particular, the NECA HTR program is a unique initiative in the Asian region, benefitting from dedicated financial investment by the government. Despite lacking a specific supporting legislation, the HTR program’s rapid expansion and comprehensive coverage can be attributed to both the political will to achieve universal healthcare and a substantial government investment (9).

The primary objective of this study is to introduce a comprehensive implementation process and functional methods for HTR, as implemented by NECA, offering an overview of how this program works with stakeholders in the Korean context.

## International trends in HTR

HTR systems have been established worldwide since the mid-2000s. While Spain and France operate separate HTR programs, other countries, such as the United Kingdom (UK) and Australia, operate reassessment systems within the HTA process (10;11). Of these four, only France conducts a regular review of publicly funded technologies as a political HTR. In the other three countries (UK, Australia, and Spain), HTR is conducted on request by authorities (10). While HTR could be used only to decide disinvestment in some countries, most countries, including Korea, aim to reallocate health resources and optimize the use of existing health technologies, as appropriate.

Generally, HTA agencies conduct reassessments with processes and methods that are not significantly different from those of HTA. The relevant bodies assess clinical safety, effectiveness, and cost-effectiveness based on systematic reviews and use other methods such as cost-effectiveness analysis, investigations of medical use amount, and patients’ preferences in their country. For HTR, social and ethical factors should be assessed more comprehensively in addition to clinical and cost-effectiveness parameters involving various stakeholders. The results of the HTR are presented as recommendations and used as a basis for decision-making by policy-making authorities (regarding issues such as public resource investments and benefits) and stakeholders, such as healthcare providers and users.

The involvement of stakeholders throughout the process and exchanges of perspectives or preferences are important for successful implementation of HTR recommendation (3) and for the desired goal achievement (12). Continuous knowledge exchange is important throughout the HTR process, and stakeholders are involved in the selection, prioritization, and identification of reassessment topics, research question developments, knowledge generation, and interpretation of findings (3;12).

## HTR committee

The HTR committee in NECA was formed in 2019, operating separately from the new HTA (nHTA) due to the differences in processes and content. The HTR committee serves as an advisory body to NECA, providing recommendations related to existing technologies through HTR processes. These recommendations are crucial for informing NECA’s key customers and stakeholders.

The HTR committee comprises 1 chairman and 18 members and recruits various professionals, including individuals from medical, dental, and traditional Korean medicine societies, legal experts (lawyers), health economists, HTA specialists, as well as representatives of patients, consumers, and civic groups, and the government. Transparency and ethical conduct are secured, requiring committee members to declare any conflicts of interest or potential influences on HTR tasks within NECA.

The HTR committee convenes monthly focusing on evidence-based advices integrating insights from medical and scientific evidence, ongoing clinical practices, economic considerations, ethical aspects, patients’ perspectives, and social values. The holistic approach by the committee ensures a comprehensive and in-depth assessment process for HTR.

## HTR system in NECA

Since its establishment, NECA has played a crucial role in supporting the deliberations of the nHTA committee by producing evidence for new technologies beyond drugs (13;14). Recently, NECA initiated the HTR project, aligned with previous studies (1–4), to compare the effectiveness and cost-effectiveness of existing technologies and to make recommendations. It aims to facilitate the appropriate utilization of these technologies by clinicians and patients and support relevant insurance policy decisions. Ultimately, this initiative aims to optimize the allocation of health resources and encourage the optimal use of health technologies.

**Figure 1.**
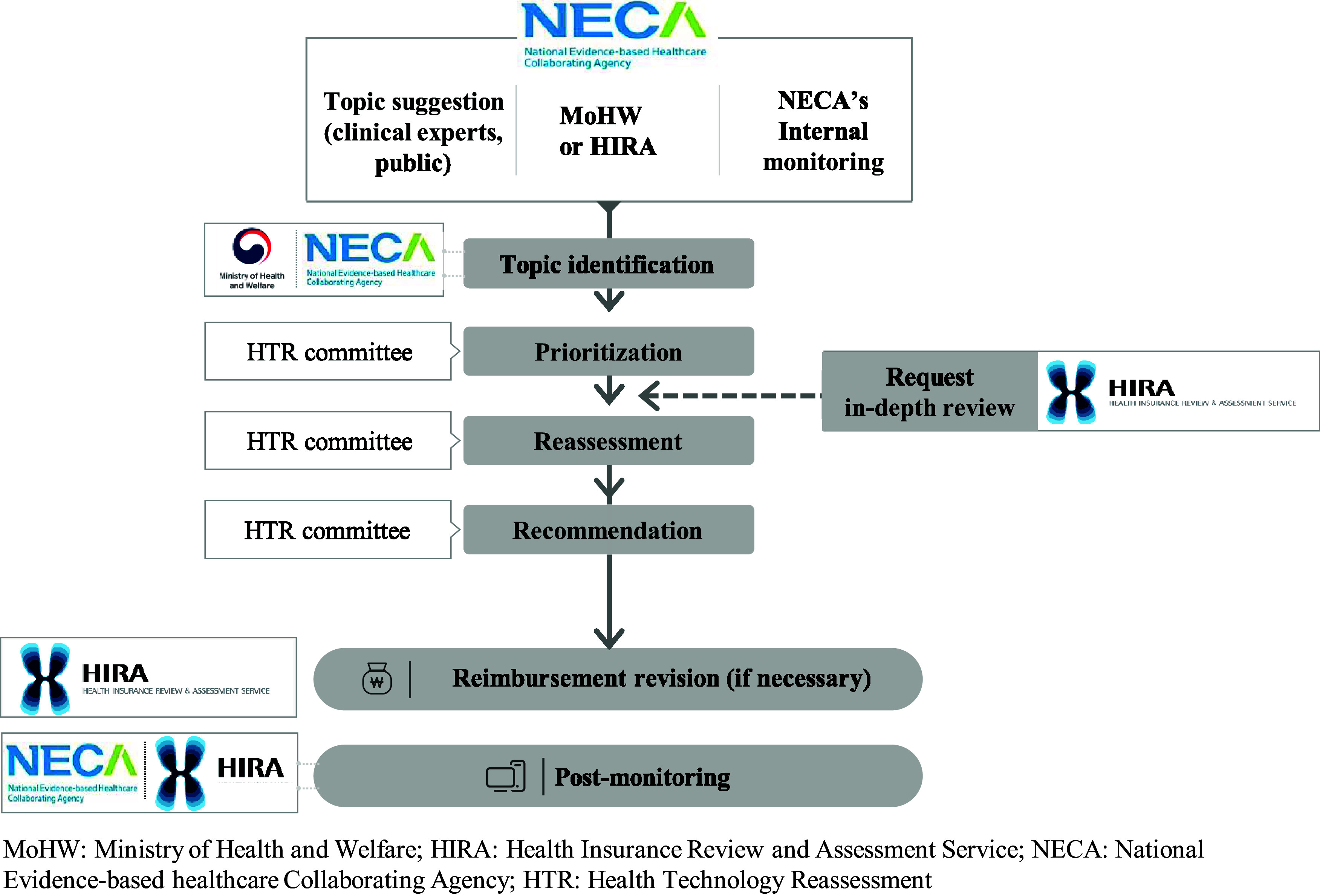
The process of health technology reassessment.

NECA’s HTR scope encompasses several technologies, including those with covered benefits and selective or preliminary benefits, and even those not covered by the NHI. The HTR committee, which is responsible for significant decisions, discusses and determines process details within the subcommittees. The HTR process is structured into three stages: topic selection, assessment, and recommendation (Figure 1).

### Topic selection for HTR

The technology selection follows a structured three-step process: candidate listing, screening, and prioritization. The subjects of reassessment are health technologies currently used in the Korean clinical practice, with concerns related to safety, uncertain effectiveness, or unexpected changes in use and healthcare costs within the past 5 years.

The process of listing candidates includes external topic suggestions from the medical society, related governmental organizations, public involvement groups, and internal monitoring activities. Anyone, including stakeholders, decision-makers, healthcare experts, and patients, can propose topics for reassessment via email using a submission form. Furthermore, internal monitoring is conducted through strategies tailored and established for each type of NHI reimbursement (Table 1).

**Table 1.** Internal monitoring strategy of candidate HTR topic by reimbursement type

Type of reimbursement	Internal monitoring strategy
Services registered in the benefit list and covered by NHI	Health technologies not recommended by clinical guidelines (e.g., NICE do not do recommendations, choosing wisely, etc.) Health technologies that already have many alternatives (reduced clinical usage) Health technologies listed on NHI prior to the nHTA system introduction in Korea (not reviewed through an HTA process) Topic suggestion (clinical experts or other stakeholders)
Service registered in the benefit list and partially covered by NH (preliminary benefits)	Screening items subject to next year’s suitability assessment program
Services registered in the benefit list but non–covered by NHI	Items related to NHI coverage expansion plan Topic suggestion (clinical experts or other stakeholders)
Services considered medically unnecessary (e.g., health screening test, cosmetic and plastic surgeries, etc.)	Items for cost investigation in clinical fields (report from non–covered task force) Topic suggestion (patients or other stakeholders)

Abbreviations: NICE, National Institute for Health and Clinical Excellence; nHTA, new Health Technology Assessment; NHI, National Health Insurance.

The screening step involves a preliminary review of candidate technologies, where NECA’s HTR staff develops an information sheet containing current evidence and usage data in Korea. This information is provided to support the candidate selection, which is then evaluated by clinical experts. Experts within the relevant clinical fields assess whether the topic is suitable for in-depth reassessment. The following health technologies are deemed unsuitable for HTR: (i) not approved by the Ministry of Food and Drug Safety, (ii) not registered in the NHI list, and (iii) without alternative treatments for rare incurable disease. If more than one-third of the attending members determine a topic as unsuitable for HTR, it will be excluded in the screening stage.

The final stage of topic selection is prioritization by the HTR committee, emphasizing procedural transparency. The selection criteria encompass evaluating the need for safety, clinical effectiveness, cost-effectiveness, social impact (including health and equity), and feasibility of reassessment. Topics that score 70 or more out of 100 are chosen for HTR.

### Evidence assessment of health technologies

The reassessment process is systematically structured, involving a planning stage to decide the scope of reassessment, actual reassessment, and preparation of a comprehensive report. The final scope and methodology of reassessment are approved by the HTR committee.

The assessed scopes are mainly categorized into (i) clinical usage; (ii) safety and effectiveness; (iii) cost-effectiveness; and (iv) social value (patient preference, health impact, or equity). Safety and effectiveness are the main domains assessed through systematic reviews and outcomes research studies. Cost-effectiveness and social value are optionally reviewed, depending on reimbursement type, disease burden, and social need. Literature regarding cost-effectiveness analysis is reviewed based on the reimbursement type (health services covered by the NHI or preliminary benefit with 50 percent copayment). The preliminary benefit with 50 percent copayment is provided when the medical necessity and effectiveness are confirmed but cost-effectiveness is uncertain when reimbursement is listed. Furthermore, a separate economic evaluation is conducted when additional evidence are required for cost-effectiveness in Korean context.

Focus group interview (FGI) with patients or survey for public involvement in NECA (PIN) is a crucial step in evaluating the social value. Patient’s FGI considers the disease burden as well as social impact of the health technology. However, the survey for PIN, grounded on evidence regarding safety, effectiveness, and other relevant factors, helps evaluate the acceptability of the health technology. Additionally, a budget impact analysis is performed to predict the potential impact of the technology on the NHI budget in the next 5 years. It considers the application of new health insurance benefits or changes in the current benefit criteria.

In NECA’s HTR process, real-world evidence (RWE) is not the primary focus and has been applied only in a few cases. NHI claims data are the main source for HTR within NECA. The claims data sets exhibit high completeness for variables necessary for the evaluation of technologies registered in the NHI list and include medical cost, utilization, and the characteristics of the population using those technologies. A major strength of insurance or medical record databases is the extensive data from nearly 100 percent of Korean population with specific conditions (15;16).

In 2020, the NECA HTR started a pilot project that reflected the social value evaluated by the public. This group is comprised of individuals selected for PIN or patients involved in the decision-making process for the recommendation system. The NECA’s reassessment execution staff conducts the evidence review according to the approved protocol, and subcommittee meetings are held at each stage. The report is developed by integrating information about a technology’s background, disease indication, clinical safety, effectiveness, cost-effectiveness analysis, and social value with the subcommittee’s review. A draft report is submitted to the HTR committee.

### Recommendations

To determine the recommendations for HTR to promote the efficient use of healthcare resources, NECA strives to establish a scientifically robust and practical system for achieving consensus. The existing recommendation system, based on the Grading of Recommendation, Assessment, Development and Evaluation system, comprises four grades (Table 2): “recommended,” “conditionally recommended,” “not recommended,” and “insufficient.” The HTR committee decides the recommendations, and agreements are discussed during meetings.

**Table 2.** Recommendation system of HTR project

	Explanation
Recommended	Considering that the clinical safety and effectiveness of the relevant health technology are sufficient and other aspects (cost–effectiveness, value, etc.), the use of it is recommended in most domestic clinical situations.
Conditionally recommended	Considering that the clinical safety and effectiveness of the relevant health technology are sufficient and other aspects (cost–effectiveness, value, etc.), its clinical use may vary depending on the clinical situation or preference; therefore, it is recommended under conditions or restrictions.
Not recommended	Considering that the clinical safety and effectiveness of the relevant health technology are sufficient and other aspects (cost–effectiveness, value, etc.), the use of it is not recommended in most domestic clinical situations.
Insufficient	Due to insufficient clinical evidence to determine the clinical safety and effectiveness of the relevant health technology, the recommendation for its use cannot be determined in domestic clinical situations. ※ Health technology that has been deliberated as “insufficient” may be mentioned in a decision letter containing the reasons for the decision and HTR’s follow–up plan.

Abbreviation: HTR, Health Technology Reassessment.

## Performances and impacts in NECA’s HTR

Since its pilot launch in 2018 up to 2023, 262 cases have been reassessed (Table 3). Of these, 126 (48.1 percent) were not covered by the NHI and a high proportion were aimed at supporting the plan for benefit expansion in the NHI. Over 5 years, the HTR committee has formulated approximately 130 recommendations for currently used technologies.

**Table 3.** NECA’s HTR performance over the past 5 years

Type of reimbursement	2018	2019	2020	2021	2022	2023	Total
Services registered in the benefit list and covered by NHI	2	4	17	3	6	20	52 (19.8%)
Service registered in the benefit list and partially covered by NH (preliminary benefits)	5	5	6	7	18	21	62 (23.7%)
Services registered in the benefit list but non–covered by NHI	8	24	26	37	24	7	126 (48.1%)
Services considered medically unnecessary (e.g., health screening test, cosmetic and plastic surgeries, etc.)	2	6	4	4	4	3	22 (8.4%)
Total	17	39	53	51	51	51	262 (100.0%)

Abbreviations: NECA, National Evidence-based Healthcare Collaborating Agency; HTR, Health Technology Reassessment; NHI, National Health Insurance.

In 17 cases, items transitioned from non-covered by the NHI or the range of reimbursement was reduced. Additionally, five cases were removed from the list of non-covered items due to non-use in the clinical field. Forty-seven HTR reports were submitted to the MoHW and Health Insurance Review and Assessment Service (HIRA) for their review and consideration. Moreover, public information or knowledge transfer activities were conducted to assist patients and medical service consumers in making informed and rational choice. The following representative cases of HTR were outlined.

### Case 1: Robot-assisted gait training

Robot-assisted gait training (RAGT)-based rehabilitation is within “Gait Training (Sa130, charge code MM302)” category and is covered by the NHI. This technology is widely used in physiotherapy and has demonstrates superior clinical outcomes compared to conventional rehabilitation. Based on this intent, both the manufacturers and clinical experts of RAGT technology have advocated an increase in the NHI charge for RAGT-based rehabilitation. In response, the HIRA requested the HTR committee to reassess whether RAGT was more clinically effective than conventional rehabilitation.

A systematic literature review was conducted encompassing all diseases eligible for reimbursement indications, such as quadriplegia, Parkinson’s disease, amputation, peripheral nervous system disease, progressive muscular dystrophy, traumatic brain injury, and stroke. Based on the level of evidence derived from this review, the HTR committee concluded that RAGT-based rehabilitation was a safe technique to be used for patients with stroke, Parkinson’s disease, multiple sclerosis, or spinal cord injury. Furthermore, the committee found no significant difference in clinical effectiveness compared to that of conventional therapies in aspects such as gait, balance, quality of life, and fatigue. Consequently, the HTR committee conditionally recommended RAGT for the rehabilitation of patients with stroke, Parkinson’s disease, multiple sclerosis, and spinal cord injury and pediatric patients. However, for other indications, including lower-limb amputation, traumatic brain injury, and lower-limb burns, the HTR committee deemed the evidence to be insufficient for a recommendation.

After discussing the HTR results, the MoHW decided to implement a preliminary benefit in 2022 for RAGT especially for patients with stroke. Under this provision, patients are required to make a 50 percent copayment for the RAGT service.

### Case 2: Hemoperfusion with a polymyxin B-immobilized fiber column

Hemoperfusion with a polymyxin B-immobilized fiber column (PMX-DHP) is a direct extracorporeal hemoperfusion technique that employs a column containing polymyxin B, an antibiotic, to eliminate endotoxins from the blood of patients experiencing sepsis or septic shock.

In 2019, owing to insufficient clinical evidence and high treatment costs borne by patients with severe conditions, PMX-DHP was designated as a preliminary benefit with a 90 percent copayment. Subsequently, the HIRA requested the HTR committee to conduct a reassessment focusing on the safety and effectiveness of PMX-DHP.

Based on the evidence from a systematic review, the HTR committee concluded that PMX-DHP is safe, with rare complications. However, in terms of effectiveness, it did not yield improvement in the mortality rate, which is a critical outcome, at any of the tested time points or according to the Multiple Organ Dysfunction Score. Consequently, the HTR committee did not recommend the utilization of PMX-DHP for patients with sepsis or septic shock.

After discussing the HTR results, the MoHW decided to remove PMX-DHP from a preliminary benefit list, which previously had a 90 percent copayment. Instead, it was relisted as a non-covered item in 2022.

### Case 3: Hyperthermia combined with radiation therapy

Hyperthermia combined with radiation therapy is a cancer treatment aimed at eradicating cancer cells while minimizing harm to normal tissues. This technique involves subjecting body tissues to elevated temperatures (up to 45 °C), causing damage or destruction of cancer cells or rendering them more susceptible to radiation or chemotherapy. Despite its potential, hyperthermia combined with radiation therapy has been considered as a non-covered item by the NHI since January 2005, and the HIRA requested a reassessment to gather evidence and evaluate the possibility of expanding insurance coverage.

Based on the evidence derived from a systematic review, the HTR committee concluded that hyperthermia can be an adjuvant treatment alongside radiation therapy and/or chemotherapy. However, the evidence was deemed insufficient, and additional therapeutic effects could not be confirmed even when some evidence was presented contingent upon specific types of cancer.

The HTR committee concluded that the combination of hyperthermia and radiation therapy in patients with cancer was not recommended for coverage by NHI due to its lack of effectiveness, despite being used for over 10 years.

### Case 4: Saw palmetto

Saw palmetto (*Serenoa repens*) is a fruit extract of saw palm trees, often marketed to alleviate urination symptoms. Saw palmetto extract was considered as a topic owing to the interest of the PIN group due to its widespread exposure in the media and advertisements. Consequently, the HTR committee undertook the task of providing public information concerning the safety and effectiveness of saw palmetto extracts in treating prostate hypertrophy.

After assessing the evidence through an overview of the systematic review, the HTR committee concluded that saw palmetto extract poses no safety concerns for benign prostatic hyperplasia. However, the evidence could not substantiate the improvement in prostatic hyperplasia. The outcomes of HTR were consolidated and information were provided to the public to promote rational health consumption and support informed decision-making.

## Discussion

The ultimate goal of treatment is to maximize health benefit, encompassing disease treatment, lifespan extension, and improvements in overall quality of life. However, the increased prevalence of chronic diseases, exacerbated by demographic factors, such as population aging, is accelerating this trend and significantly straining healthcare budgets (13). Given the constraints of limited resources and the increasing costs associated with health technologies, there is a pressing challenge to prioritize and optimize net benefits. HTA plays a vital role in evaluating the benefits of these technologies for both patients and healthcare systems. It aids in informing health policy decision-making regarding the utilization of existing health technologies.

In Korea, there has been a growing need for health policy reforms and strategies that promote effective management and appropriate use of existing health technologies, considering their evolving value throughout the life cycle. To address this need, NECA has established an HTR system that aims to effectively manage the life cycle of health technologies by updating evidence based on existing technologies. The technologies include those for which nHTA has been conducted, enabling NECA to conduct reassessments based on up-to-date evidence. This study introduced the recent activities undertaken by NECA in executing the HTR with the primary goal of promoting the optimal use of health technologies and supporting affordable healthcare coverage and decision-making. The selection of NECA HTR items is based on the evaluation of various factors such as safety, effectiveness, cost-effectiveness, social impact, and feasibility. Recommendations are based on the assessment results and perspectives of stakeholders, including clinical experts, the public, and patients, at every stage of the process.

Specifically, the NECA intends to improve the HTR system to enhance objectivity and maintain consistency in reassessment. This is to ensure adequate consideration of values for the public and patients within the context of social values. In 2018, NECA initiated the PIN system, and the ongoing third-term PIN commenced in 2022 comprises more than 100 Korean residents aged over 19 years. This group was formed to facilitate rational decision-making considering social values. The PIN serves as a mechanism for public involvement, enabling patients and the public to have their thoughts and values integrated into the HTA process (17). Individuals participating in the PIN can share their experiences and opinions as medical users through various activities. They can contribute at any point in the HTR process, including suggesting topics, defining assessment scopes, incorporating social values during recommendations, and aiding in knowledge transfer. It is noteworthy that NECA’s HTR initiative not only informs health insurance benefit determination but also emphasizes the public’s right to information access. Through the dissemination of health knowledge, it strives to facilitate shared decision-making among the public.

Well-designed and effectively executed deliberative processes are crucial in enhancing public trust and ensuring the legitimacy of decisions (18). Consequently, utilizing evidence in systematic reviews helps substantiate the use of values and resources to mitigate subjectivity (19). In many cases, institutions responsible for conducting HTA at the national level operate independently from the competent authorities they serve (e.g., ministries of health and health insurance organizations). However, these activities may be supervised by these authorities (20).

Since the pilot in 2018, NECA has been actively developing the HTR system; however, there are areas to improve. Initially, the HTR focused on non-covered items with a high proportion of support policies to strengthen the NHI coverage; however, NECA aims to enhance the reassessment of the effectiveness and cost-effectiveness of covered items. In particular, technologies that were reimbursed before the introduction of the nHTA system have not been assessed on safety and effectiveness. Due to the rapid advancement of health technologies and the complexity in clinical practice, evidence-based policymaking regarding resource allocation, rationing, and priority setting increasingly demands explicit justification of decisions. Policymakers face the challenges of balancing short-term and long-term costs and gains, alongside consideration of social values, cultural beliefs, uncertainty, risk perceptions, politics, ideology, and the political economy, all of which extend beyond empirical evidence. In this context, it is crucial to focus on how evidence-based interventions are effectively transferred to diverse implementation sites (21), highlighting the necessity for continuous reassessment. There is a call to officially develop and establish an HTR system with a solid legal foundation to reoptimize the NHI system and ensure efficient utilization of healthcare resources.

The incorporation of RWE in HTR significantly aids decision-makers in the efficient allocation of healthcare resources, facilitating reinvestment or disinvestment in health technologies (22;23). However, within the NECA, the NHI claims data are the primary source for HTR, limiting the application of RWE to services covered by the NHI. Recognizing the limitations of insurance claims data due to its administrative nature (15;24), the NECA must enhance its framework to effectively utilize RWE in assessing the effectiveness and cost-effectiveness within HTR. Furthermore, the MOHW is considering a new system for generating evidence for reimbursement-related decision-making. This initiative will strengthen the role and impact of RWE in Korea’s HTR.

Finally, monitoring and evaluating the outcomes of HTR are critical to assess whether program has achieved its intended objectives. This monitoring process also serves as the initial step in identifying health technologies that require reassessment and further informs the HTR process. Currently, we are actively striving to monitor the impact of HTR recommendations by utilizing real-world data such as claims data, changing statement of clinical guidelines, or surveying public perspectives. Although there is time gap between HTR decision and practice changes in real world, we recognize the significance of this task and are committed to its effective implementation.
